# Aberrant activation of hedgehog signaling promotes cell proliferation via the transcriptional activation of forkhead Box M1 in colorectal cancer cells

**DOI:** 10.1186/s13046-017-0491-7

**Published:** 2017-02-02

**Authors:** DeJie Wang, Guohui Hu, Ying Du, Cheng Zhang, Quqin Lu, Nonghua Lv, Shiwen Luo

**Affiliations:** 1 Institute of Digestive Disease, The First Affiliated Hospital of Nanchang University, 17 Yongwai Street, Donghu District, Nanchang, Jiangxi 330006 China; 2 Center for Experimental Medicine, The First Affiliated Hospital of Nanchang University, Nanchang, Jiangxi 330006 China; 3 Department of Epidemiology & Biostatistics, School of Public Health, Nanchang University, Nanchang, Jiangxi 330006 China; 4 Jiangxi Key Laboratory of Molecular Diagnosis and Precision Medicine, Nancahng, Jiangxi 330006 China

**Keywords:** Hedgehog, Gli1, FoxM1, Colorectal cancer, Proliferation

## Abstract

**Background:**

Recent evidence suggests that the aberrant activation of Hedgehog (Hh) signaling by Gli transcription factors is characteristic of a variety of aggressive human carcinomas, including colorectal cancer (CRC). Forkhead box M1 (FoxM1) controls the expression of a number of cell cycle regulatory proteins, and FoxM1 expression is elevated in a broad range of human malignancies, which suggests that it plays a crucial role in tumorigenesis. However, the mechanisms underlying FoxM1 expression are not fully understood. Here, we aim to further investigate the molecular mechanism by which Gli1 regulates FoxM1 in CRC.

**Methods:**

Western blotting and immunohistochemistry (IHC) were used to evaluate FoxM1 and Gli1 protein expression, respectively, in CRC tissues and matched adjacent normal mucosa. BrdU (5-bromo-2′-deoxyuridine) and clone formation assays were used to clarify the influence of FoxM1 on CRC cell growth and proliferation. Chromatin immunoprecipitation (ChIP) and luciferase experiments were performed to explore the potential mechanisms by which Gli1 regulates FoxM1. Additionally, the protein and mRNA expression levels of Gli1 and FoxM1 in six CRC cell lines were measured using Western blotting and real-time PCR. Finally, the effect of Hh signaling on the expression of FoxM1 was studied in cell biology experiments, and the effects of Hh signaling activation and FoxM1 inhibition on the distribution of CRC cells among cell cycle phases was assessed by flow cytometry.

**Results:**

Gli1 and FoxM1 were abnormally elevated in human CRC tissues compared with matched adjacent normal mucosa samples, and FoxM1 is a downstream target gene of the transcription factor Gli1 in CRC and promoted CRC cell growth and proliferation. Moreover, the aberrant activation of Hh signaling promoted CRC cell proliferation by directly binding to the promoter of FoxM1 and transactivating the activity of FoxM1 in CRC cells.

**Conclusion:**

The dysregulation of the Hh-Gli1-FoxM1 axis is essential for the proliferation and growth of human CRC cells and offers a potent target for therapeutic intervention in CRC.

**Electronic supplementary material:**

The online version of this article (doi:10.1186/s13046-017-0491-7) contains supplementary material, which is available to authorized users.

## Background

Colorectal cancer (CRC), a common malignant tumor of the digestive tract, is one of the leading causes of cancer death in both developed and developing nations. This disease has an estimated annual worldwide incidence of more than one million new cases, and approximately one of every three people who develop CRC dies from the disease [1]. The current treatment for CRC patients is mainly based on comprehensive surgical treatment with chemotherapy and/or targeting therapies. Although the molecular mechanisms of CRC development and progression have been extensively researched, the prognosis of patients with CRC remains unsatisfactory, especially for patients with lymph node metastases [2]. Therefore, a better understanding of the molecular mechanisms of CRC tumorigenesis and the development of new therapeutic targets based on these mechanisms are of great significance.

The zinc-finger transcription factor Gli1 is a key downstream effector of the Hedgehog (Hh) signaling pathway, which functions via a membrane-protein complex that consists of Patched-1 (Ptch1) and Smoothened (Smo) [3]. Physiologically, the activation of Hh signaling is initiated by the binding of the Hh ligand to the Ptch1 receptor. As a result of this binding, Smo is activated, which consequently activates transcription factor Glis. Three Gli proteins are known, and they exert both activator and repressor functions. Specifically, Gli1 acts as a transcriptional activator, Gli2 is a composite of positive and negative regulatory domains, and Gli3 acts primarily as a transcriptional repressor [4]. The activated Gli proteins translocate to the nucleus and transactivate many downstream target genes, such as Gli1 itself, Ptch1, Cyclin D1, p21 and Snail [5]. The aberrant activation of Hh-Gli signaling has been implicated in the promotion of tumorigenesis in several types of carcinoma, including hepatocellular carcinoma [6], gastric cancer [7, 8], lung cancer [9] and basal cell carcinomas [10]. Similar results were also reported in other studies of CRC [11–13]. Although these authors found that the Gli1 mRNA and protein expression levels were significantly increased in CRC tissues, the exact mechanism underlying this increase remained unclear. Therefore, the molecular mechanisms by which the aberrant activation of Hh signaling promotes CRC cell proliferation and tumor growth need to be further elucidated.

Forkhead box M1 (FoxM1) is a transcription factor of the forkhead family, which consists of more than 50 transcription factors that share a conserved forkhead or winged-helix DNA-binding domain [14]. FoxM1 is expressed in embryonic tissues and dividing cells of epithelial and mesenchymal origin, but not in terminally differentiated, non-dividing cells [15]. FoxM1 plays a critical role in cell cycle progression. Specifically, the expression of FoxM1 increases at the G1 to S phase transition and reaches a maximal level during the G2 to M phase transition, thereby promoting M phase entry [16, 17]. FoxM1 controls the expression of a number of cell cycle regulatory proteins, including cyclin B1 [17], and genes that are essential for faithful chromosome segregation and mitosis, such as Cdc25B, Aurora B kinase, Survivin, PLK1, centromere protein A (CENPA), and CENPB [16, 18]. Moreover, FoxM1 has been described to be involved in a broad range of human malignancies [19–22]. Recently, Zhang et al. found that the overexpression of FOXM1 contributed to the progression of CRC [23]. Furthermore, another study indicated that FoxM1D promoted epithelial-mesenchymal transition and metastasis by interacting with ROCK2 in CRC [24]. However, the molecular mechanisms by which FoxM1 promotes CRC cell proliferation have not been fully elucidated.

In our previous gene expression profile analysis (GSE54936 and GSE53464) [25, 26], FoxM1 was downregulated in human glioma and ovarian cancer cells after treatment with the Hh-Gli signaling pathway inhibitor GANT61 [27, 28]. Thus, we speculate that Gli1 promotes CRC cell proliferation by regulating FoxM1 expression. To further explore the mechanisms by which Gli1 regulates FoxM1, we constructed ChIP and luciferase reporter assays in this study and identified FoxM1 as a downstream target gene of Gli1 in CRC. Our results provide evidence that Gli1 transcriptionally activates FoxM1 expression by directly binding to the promotor of FoxM1. We also show that Gli1 promotes the proliferation of CRC cells by transactivating FoxM1 and upregulating the expression of FoxM1.

## Methods

### Cell culture, small molecular reagents and constructs

HEK293T and six CRC cell lines (HT-29, HCT116, LoVo, Caco-2, SW620 and SW480) were obtained from the American Type Culture Collection (ATCC, Manassas, VA). HEK293T cells were cultured in basal Dulbecco’s Modified Eagle Medium. The basal medium for the HT-29 and HCT116 cell lines was ATCC-formulated Modified McCoy’s 5α Medium; the basal media for LoVo and Caco-2 cells were ATCC-formulated F-12 K Medium and Eagle’s Minimum Essential Medium, respectively; and Leibovitz’s L-15 Medium was used for SW620 and SW480 cells. Each basal medium was supplemented with 10% fetal bovine serum (Gibco-Life Technologies, Grand Island, NY). The cell lines were maintained at 37 °C in a humidified atmosphere containing 5% CO_2_. The small molecular regents were obtained from the following sources: purmorphamine (Selleck Chemicals, Houston, TX), GANT61 (Sigma-Aldrich, St. Louis, MO), thiostrepton (Sigma-Aldrich, St. Louis, MO), and cyclopamine (Sigma-Aldrich, St. Louis, MO); DMSO was used as the solvent for these regents and the vehicle control.

The human full-length FoxM1 (NM_202002) construct was subcloned into pcDNA3.1-Myc/His (Invitrogen, Carlsbad, CA). The human full-length Gli1 (NM_005269) construct was subcloned into pUB6-V5/hisB (Invitrogen, Carlsbad, CA). The miRNAi-FoxM1 expression vectors that suppress FoxM1 expression and the miRNAi-Gli1 expression vector were generated using the BLOCK-iT™Pol II miR-RNAi Expression Vector System (K4936-00, Invitrogen, Carlsbad, CA) as discribed earlier [29, 30]. Briefly, based on an analysis of the human FoxM1 and Gli1 sequences using a program provided by Invitrogen, three regions were cloned into the pcDNA™6.2-GW/EmGFP-miR expression vectors to yield miRNAi-FoxM1 or miRNAi-Gli1. They were co-transfected with FoxM1/Gli1 expression construct into HEK293T cells to identify effective clones based on their ability to suppress FoxM1/Gli1 expression by Western blotting analysis. The following oligonucleotide sequences were used to generate the miRNAi constructs: for miRNAi-FoxM1-1692 (targeting nucleotides 1692 to 1712 of FoxM1), 5′ -CTC TTT CTT CTG CAG GAC CAG -3′, and for miRNAi-Gli1-2855 (targeting nucleotides 2855 to 2875 of Gli1, 5′-AGA GTC CCA AGT TTC TGG GGG-3′. The authenticity of all constructs was verified by DNA sequencing.

### Western blotting and antibodies

Cells were harvested by trypsinization, lysed in 1× sodium dodecyl sulfate lysis buffer, and denatured for 10 min at 100 °C. After immunoblotting, the membranes were blocked with 5% nonfat dry milk in TBS/0.1% Tween-20 and then incubated with the primary antibodies in 1% nonfat dry milk in TBS/0.1% Tween-20. Subsequently, the blots were incubated with goat anti-rabbit or anti-mouse secondary antibody (Invitrogen, Carlsbad, CA) and visualized with enhanced chemiluminescence (Invitrogen, Carlsbad, CA).

The immunoreagents used for Western blotting were rabbit polyclonal antibody against Gli1 (Abcam, ab92611, diluted 1:500) and rabbit polyclonal anti-FoxM1 (Abcam, ab137647, diluted 1:500). Mouse monoclonal antibody against CCNB1 was purchased from Cell Signaling Technology (CST, 4135, diluted 1:2000), and anti-β-actin antibody (Anbo, E0012, diluted 1:5000) or anti-GAPDH antibody (Millipore, MAB374, diluted 1:2000) was used as a loading control.

### Immunohistochemistry

First, 3-μm-thick CRC tissue sections were deparaffinized, rehydrated, and treated with 3% H_2_O_2_ to block endogenous peroxidase activity. After the sections were pretreated for antigen retrieval by microwaving them in ethylenediamine tetraacetic acid (EDTA) (pH 9.0) for 25 min, they were rinsed in phosphate-buffered saline (PBS) and incubated with various primary antibodies overnight at 4 °C in a humidified chamber. The next morning, the slides were rinsed with PBS and then incubated for 40 min at 37 °C with the appropriate biotinylated immunoglobulins (Zhongshan Biotechnology, China) before visualizing the immunoreactivity using a Polink-2 HRP DAB Detection kit (Zhongshan Biotechnology, China) following the manufacturer’s protocol. Negative controls were performed in each case by replacing the primary antibody with normal IgG. The following primary antibodies were used: anti-Gli1 (Abcam, ab92611, diluted 1:100) and anti-FoxM1 (Santa Cruz, SC-502, diluted 1:300). An FSX100 microscope equipped with a digital camera system (Olympus, Japan) was used to obtain the immunohistochemistry images.

### Real-time PCR

Total RNA was harvested from CRC cells using TRIzol Reagent (Invitrogen, Carlsbad, CA) and evaluated by real-time PCR. Briefly, 1 μg of RNA was reverse-transcribed to cDNA using the PrimeScript^®^ RT reagent Kit (Takara, Japan). To quantify the mRNA levels, cDNA was amplified by real-time PCR with the SYBR Premix Ex Taq RT-PCR kit (Takara, Japan) on an ABI StepOnePlus™ Real-Time PCR System, and GAPDH was used as the internal control. The sequences of primers used for real-time PCR are shown in Additional file 1: Table S1.

### Transient transfections and luciferase assays

The human FoxM1 promoter was amplified from a human genomic DNA template and inserted into the pGL4.20 basic vector (Promega, Madison, WI). A mutant Gli1 binding motif was generated using a PCR mutagenesis kit (ToYoBo, Japan) with the forward primer (mutation sites underlined) 5′- ACA CAC CCA CGC GGC GGG GAC CCC T-3′ and a reverse complement primer. Transient transfections were performed using Lipofectamine 2000 (Invitrogen, Carlsbad, CA) according to the manufacturer’s protocol. For the luciferase reporter assays, cells were seeded in 24-well plates and transfected with the indicated plasmids. The luciferase activities were measured 48 h after transfection using a Dual Luciferase Reporter Assay System Kit (Promega, Madison, WI).

### Chromatin Immunoprecipitation (ChIP) Assay

HT29 cells were cross-linked with 1% formaldehyde, and the reaction was terminated by adding 0.125 M glycine. Chromatin was collected in 1 ml of IP buffer and sheared using a sonicator with a 4-mm tip probe using 10 3-s pulses (80 W, 90-s intervals) in an ice box. Soluble chromatin was immunoprecipitated with 4 μg of anti-Gli1 goat polyclonal antibody (Santa Cruz, sc-6152) or anti-Gli2 goat polyclonal antibody (Santa Cruz, sc-20290), and 4 μg of goat normal IgG (Santa Cruz, sc-2028) was added as a random control. DNA-protein immune complexes were eluted and reverse cross-linked by adding 0.2 M NaCl overnight at 65 °C, and DNA was extracted with phenol/chloroform and precipitated. The FoxM1 promoter domain containing the predicted Gli1 binding motifs was identified in immunoprecipitated DNA by PCR using four pairs of primers, whose sequences are shown in Additional file 2: Table S2.

### Cell viability, cell cycle and colony formation assays

Cell viability was measured using a modified MTT (3-(4, 5-dimethylthiazol-z-yl)-2, 5-diphenyltetrazolium bromide) assay. Briefly, 1 × 10^4^ cells were seeded in a 96-well plate, 0.5 mg/ml MTT (Sigma-Aldrich, St. Louis, MO) was added to each well, and the absorbance of the resultant formazan blue crystals was detected at 490 nm using a microplate ELISA reader (Bio-Rad, Hercules, CA). Moreover, a cell cycle analysis was performed by flow cytometry. After trypsinization, the cells were fixed in 70% ethanol overnight at 4 °C and stained with propidium iodide (PI). For the colony formation assay, HCT116 and Caco2 cells were seeded at the same density in 6-well dishes (2 × 10^3^ cells/well). After 20 h, the cells were treated with a different inhibitor or activator or were transiently transfected with myc-FoxM1, miR-FoxM1 or control vector. Transfectants were selected using blasticidin (4 μg/ml) for 2 weeks and stained with crystal violet. The total number of colonies in each well from three independent treatments was counted.

### Cell proliferation assay

Cell proliferation was assessed using a BrdU assay. Briefly, CRC cells were seeded in 6-well plates (2 × 10^6^ cells/well) and transfected with the miR-FoxM1, myc-FoxM1 or control vector plasmid for 24 to 48 h. After being labeled with BrdU (Sigma-Aldrich, St. Louis, MO) for 48 h, the cells were fixed and incubated with 0.5% Triton X-100 to permeabilize them. After antigen retrieval, the endogenous peroxidase activity was blocked with 3% H_2_O_2_, and the cells were then incubated with anti-BrdU antibody (Abcam, ab6326, diluted 1:40) at 4 °C overnight, followed by incubation with the appropriate biotinylated secondary antibody. Immunoreactivity was visualized using a Polink-2 HRP DAB Detection kit (Zhongshan Biotechnology, China) according the manufacturer’s protocol. Cells from the same population that were not labeled with BrdU were used as a negative cell-staining control. The relative proliferation rates are presented as percentages of the control.

### Statistical analysis

Densitometric analyses of protein bands were conducted using the ImageJ software. All data are expressed as the mean ± SD for experiments performed at least three times. Differences between 2 groups were analyzed using a two-sided paired or unpaired Student’s *t*-test. A *p*-value less than 0.05 was considered significant.

## Results

### Gli1 and FoxM1 are aberrantly elevated in human CRC tumor tissues

**Fig. 1:**
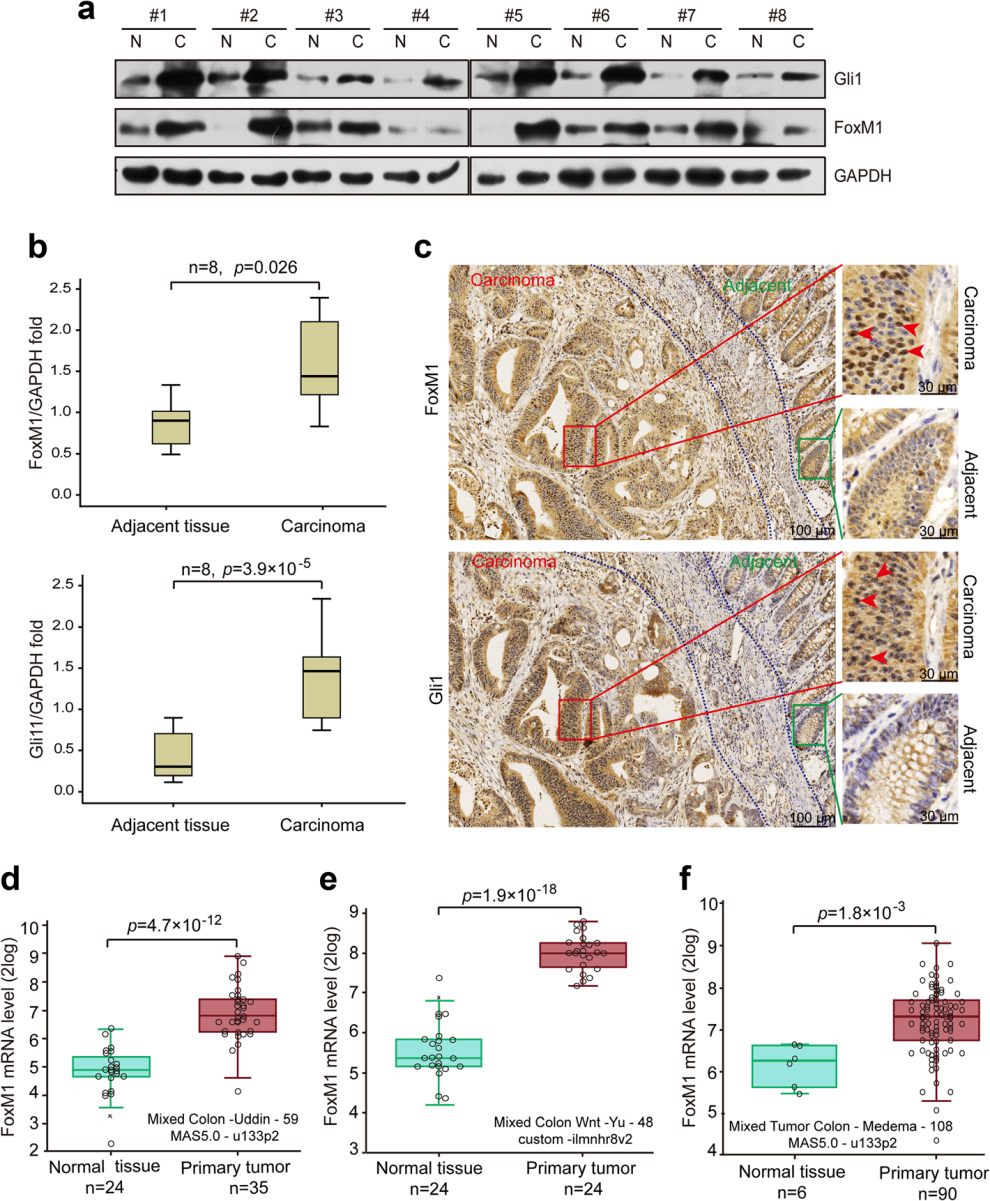
FoxM1 and Gli1 are highly expressed in CRC tissues. **a** The FoxM1 and Gli1 protein expression levels were analyzed in eight pairs of randomly selected human CRC tissues and matched adjacent non-tumor tissues by Western blotting. **b** Quantification of the Western blot (**a**). Significance was assessed using a paired samples *t*-test. **c** Immunohistochemistry staining of FoxM1 and Gli1 protein expression in a representative human CRC sample and the matched adjacent normal tissue sample in the same section. The areas of carcinoma and adjacent tissues are marked. The subcellular locations of Gli1 and FoxM1 are indicated by red arrows. **d**, **e** and **f** The mRNA expression level of FoxM1 analyzed in three published human CRC sample expression profiling studies using the R2 data sheet (http://r2.amc.nl). *p* < 0.05 was considered significant

To validate the role of FoxM1 and Gli1 in the progression of CRC, we first tested the protein levels of FoxM1 and Gli1 in primary human CRC samples and their matched adjacent normal colorectal tissues using Western blotting and immunohistochemistry analyses. Eight pairs of fresh samples were randomly collected and assessed using Western blotting, which showed that the expression levels of both FoxM1and Gli1 protein were significantly higher in the CRC tumor tissues than in their matched adjacent normal colorectal tissues (Fig. 1a and b). In line with this finding, the immunohistochemistry analysis revealed that FoxM1 and Gli1 were upregulated in carcinoma tissues compared with adjacent normal tissues (Fig. 1c). We also analyzed the mRNA expression level of FoxM1 reported in published CRC expression profiling studies using the R2 platform (http://r2.amc.nl). Supporting our results, the expression of FoxM1 was dramatically increased in CRC tumor tissues compared to normal colorectal tissues (Fig. 1d-f). Taken together, our studies demonstrate that FoxM1 and Gli1 are aberrantly elevated in CRC tumor tissues.

### FoxM1 promotes CRC cell proliferation

As a proliferation-associated transcription factor, FoxM1 plays pivotal roles in the development of various types of human malignancies, such as glioma, lung cancer, hepatocellular carcinoma, breast cancer, and pancreatic cancer [31–35]. To assess the role of FoxM1 in the progression of CRC, we examined the FoxM1 protein level in six CRC cell lines and found that his expression was lower in Caco2 cells and higher in HCT116 cells (Fig. 3a). Thus, we conducted cell colony and survival assays using the FoxM1-overexpression plasmid myc-FoxM1 in Caco2 cells (Additional file 3: Figure S1A) and an engineered miRNAi construct that efficiently reduced the expression of FoxM1 in HCT116 cells (Additional file 3: Figure S1B). As shown in Additional file 3: Figure S1C and S1D, FoxM1 overexpression increased the colony formation and growth rate of Caco2 cells, whereas knocking down FoxM1 reduced the colony formation and growth rate of HCT116 cells (Additional file 3: Figure S1E and S1F). In addition, we assessed the proliferation rate of CRC cells using a BrdU assay following treatment with the same constructs. In line with the colony formation assay, the proliferation rate of Caco2 cells increased after transfection with the myc-FoxM1 plasmid (Additional file 3: Figure S1G and S1H), and the knockdown of FoxM1 inhibited HCT116 cell proliferation (Additional file 3: Figure S1I and S1J). Taken together, these findings indicate that FoxM1 plays an important role in promoting the proliferation of CRC cells.

### Gli1 binds to the *FoxM1* promoter

**Fig. 2:**
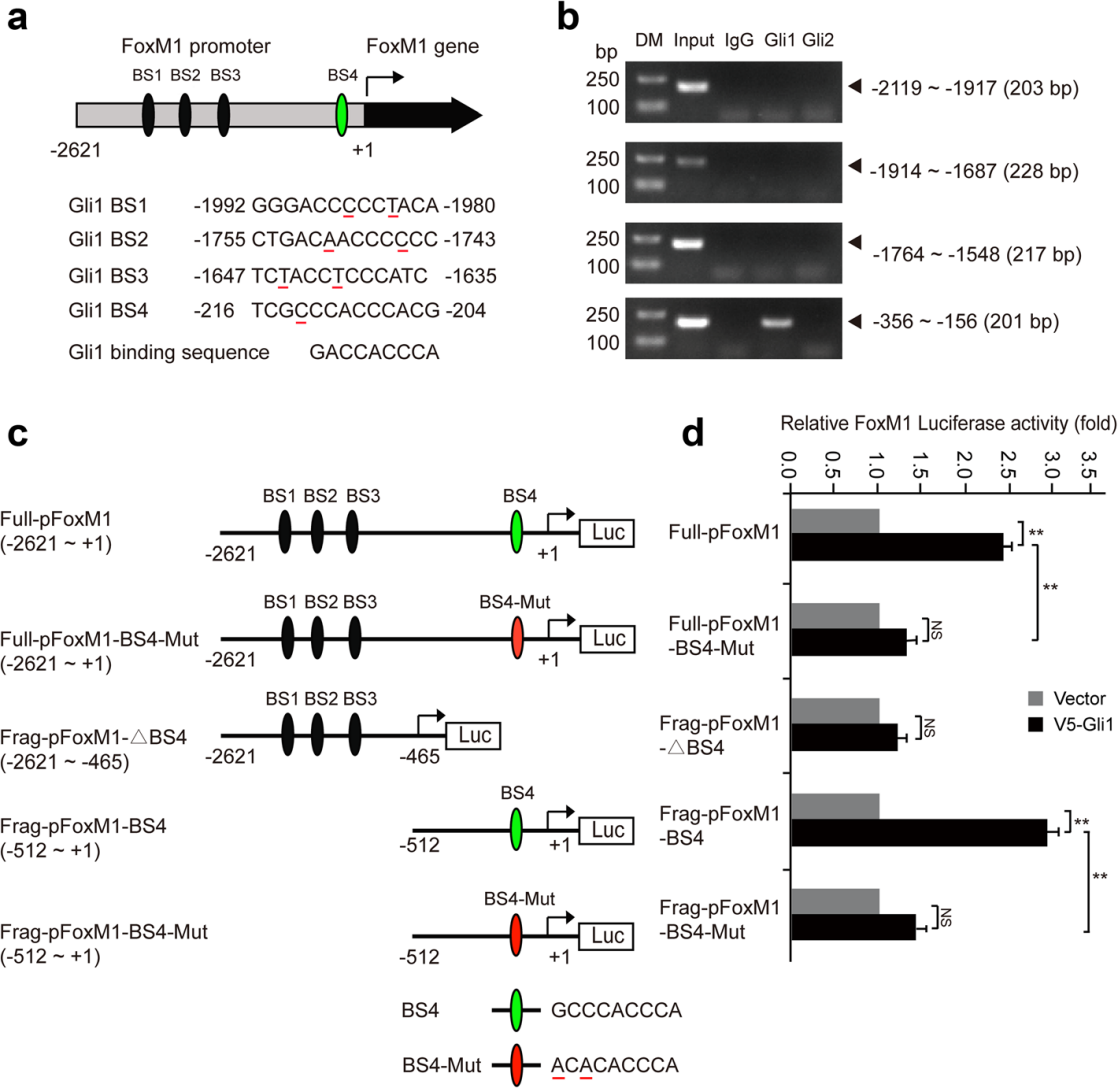
Gli1 transactivates the FoxM1 promoter. **a** Schematic diagram of four potential Gli1 binding sites (BS1, BS2, BS3, and BS4) in the FoxM1 promoter. The 9-base pair sequence of the Gli1 binding site and the sequences of four Gli1 binding sites identified in the FxoM1 promoter are shown. **b** Chromatin was isolated from HT29 cells, and ChIP assays were performed with goat IgG control, Gli1-specific and Gli2-specific antibodies. DM, DNA marker. **c** Schematic diagram of a series of FoxM1-luciferase constructs. BS4-Mut, binding site 4 mutation; △BS4, binding site 4 deletion. **d** FoxM1 luciferase constructs, as indicated, were transfected into LoVo cells together with full-length V5-Gli1 plasmid or control vector for 48 h and subjected to a luciferase reporter assay. The results were normalized to the Renilla luciferase activity and are expressed as the fold change in relative luciferase activity compared with the control. Error bars represent the mean and S.D. of three independent experiments. **, *p* < 0.01

As in our previous gene expression profile analyses (GSE54936 and GSE53464) [25, 26], FoxM1 was downregulated after the Hh-Gli signaling pathway was inhibited. In this study, we also found that FoxM1 promoted CRC cell proliferation. Thus, we hypothesized that FoxM1 is a target gene of the Hh-Gli1 signaling pathway in CRC. To determine whether Gli1 regulates FoxM1 expression by directly binding to the promoter of FoxM1, we identified four potential Gli1 binding sites (Gli1 binding motif, 5′-GACCACCCA-3′) in the gene promoter of FoxM1 using MatInspector professional version 7.2 [36]. These putative Gli1 binding sites (BS1: −1992 ~ −1980, BS2: −1755 ~ −1743, BS3: −1647 ~ −1635 and BS4: −216 ~ −204) are located upstream of the transcriptional start site of the *FoxM1* gene from −1992 bp to −204 bp (Fig. 2a). Among these four binding sites, BS1, BS2 and BS3 contained two nucleic acids that differed from the consensus sequence and shared a 78% homology with this consensus sequence, whereas BS4 exhibited only one differing base pair and shared an 89% homology with the consensus sequence. We performed ChIP studies in HT29 cells using Gli1 and Gli2, a homolog of Gli1, specific antibodies and an IgG control antibody. Although the Gli1 antibody immunoprecipitated the FoxM1 promoter containing the BS4 region, the Gli1 homolog Gli2 did not, which demonstrated that Gli1 directly bound to the FoxM1 promoter (Fig. 2b). To further confirm the role of Gli1 in the regulation of FoxM1 transcription, we generated five luciferase reporter vectors driven by the potential Gli1 binding site-containing FoxM1 promoter: Full-pFoxM1 (−2621 ~ +1), Full-pFoxM1-BS4-Mut (−2621 ~ +1-Mut), Frag-pFoxM1-△BS4 (−2621 ~ −465), Frag-pFoxM1-BS4 (−512 ~ +1) and Frag-pFoxM1-BS4-Mut (−512 ~ +1-Mut) (Fig. 2c) and performed luciferase reporter assays using LoVo cells. As expected, the overexpression of Gli1 significantly increased the luciferase activity driven by the full-length (Full-pFoxM1) or the short BS4-containing FoxM1 promoter (Frag-pFoxM1-BS4), but not the Frag-pFoxM1–△BS4 promoter, in which the Gli1 effective binding site region BS4 was deleted, or the BS4-mutated full-length FoxM1 (Full-pFoxM1-BS4-Mut) promoter (Fig. 2d). In addition, the mutated short BS4-containing promoter (Frag-pFoxM1-BS4-Mut) significantly decreased the luciferase activity compared with the Frag-pFoxM1-BS4 promoter (Fig. 2d). These results suggest that FoxM1 is a target gene of the Hh signaling pathway and that Gli1 transcriptionally activates FoxM1 by directly binding to the promoter of FoxM1 at BS4.

### Hh signaling pathway regulates the expression of FoxM1

**Fig. 3:**
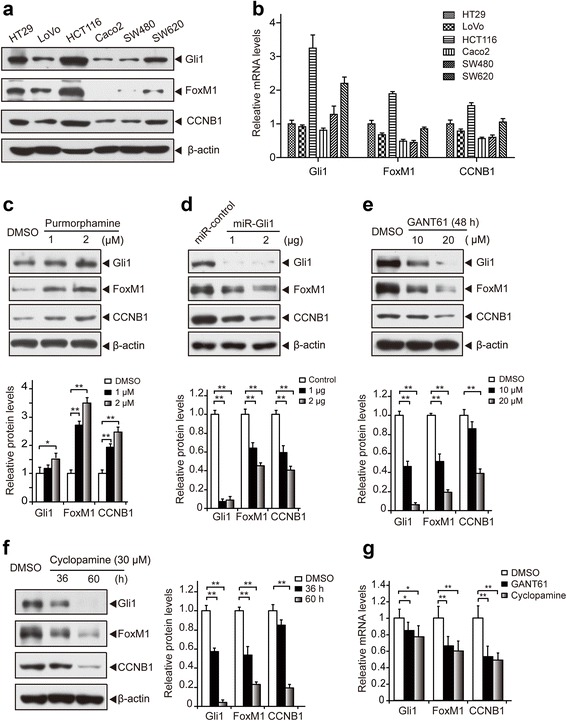
The expression of FoxM1 is regulated by Hh-Gli1 signaling. **a** Western blot analysis of the Gli1, FoxM1, and CCNB1 protein levels in six CRC cell lines. **b** Real-time PCR analysis of the Gli1, FoxM1, and CCNB1 mRNA expression levels in six CRC cell lines. The HT29 cells’ mRNA expression level was used as the normalized control. **c** Detection of Gli1, FoxM1, and CCNB1 protein expression in Caco2 cells after treatment with Hh signaling pathway activator. Caco2 cells were treated with 1 μM or 2 μM purmorphamine for 48 h, lysed and subjected to a Western blot analysis. **d**-**f** The inhibition of the Hh signaling pathway inhibited the protein expression of Gli1, FoxM1, and CCNB1, as demonstrated by the Western blot. HCT116 cells were transfected with Gli1-miRNAi or control miRNAi constructs for 48 h (**d**) or treated with the Gli inhibitor GANT61 (**e**) or Smo inhibitor cyclopamine (**f**) at the indicated time and concentrations. The cells were lysed and subjected to Western blotting. **g** Real-time PCR analysis of Gli1, FoxM1, and CCNB1 mRNA expression in HCT116 cells after treatment with GANT61 or cyclopamine. The mRNA expression levels were normalized to that of β-actin and expressed as fold change compared with the DMSO control. Error bars represent the mean and S.D. of three independent experiments. miR-Gli1: miRNAi-Gli1. *, *p* < 0.05; **, *p* < 0.01

Next, we explored the role of the Hh-Gli1 signaling pathway in the regulation of FoxM1 gene expression. To this end, we detected the expression of Gli1, FoxM1 and CCNB1 (a downstream target gene of FoxM1) [17] in several CRC cell lines by Western blotting (Fig. 3a) and real-time PCR (Fig. 3b). Although the expression levels of Gli1, FoxM1 and CCNB1 were imbalanced, both the protein levels and mRNA expression of FoxM1 and CCNB1 were consistent with Gli1 expression, indicating that the Hh-Gli1 signaling pathway likely regulated the expression of FoxM1. For further confirmation, we ectopically activated the Hh-Gli1 signaling pathway using the canonical Smo agonist purmorphamine [37]. Treating Caco2 cells with 1 μM or 2 μM purmorphamine for 48 h resulted in gradual increases in FoxM1, CCNB1 and Gli1 protein expression (Fig. 3c). We also examined the expression of FoxM1 when depleting or decreasing Gli1 expression in HCT116 cells using a Gli1 miRNAi construct or treatment with GANT61 or cyclopamine (a Smo inhibitor) [38]. The FoxM1 protein level was significantly reduced upon the depletion of Gli1 by miRNAi-Gli1 compared with the control miRNAi (Fig. 3d). Moreover, both GANT61 (10 μM or 20 μM for 48 h) and cyclopamine (30 μM for 36 h or 60 h) dramatically decreased the expression of FoxM1 (Fig. 3e and f). In line with this finding, the protein level of CCNB1 was also markedly decreased (Fig. 3d-f). Consistently, concomitant decreases in the Gli1, FoxM1 and CCNB1 mRNA levels in HCT116 cells were observed after treatment with GANT61 or cyclopamine (Fig. 3g). Overall, these data confirm that the Hh-Gli1 signaling pathway regulates the expression of FoxM1.

### FoxM1 expression is required for Gli1-mediated CRC cell proliferation

**Fig. 4:**
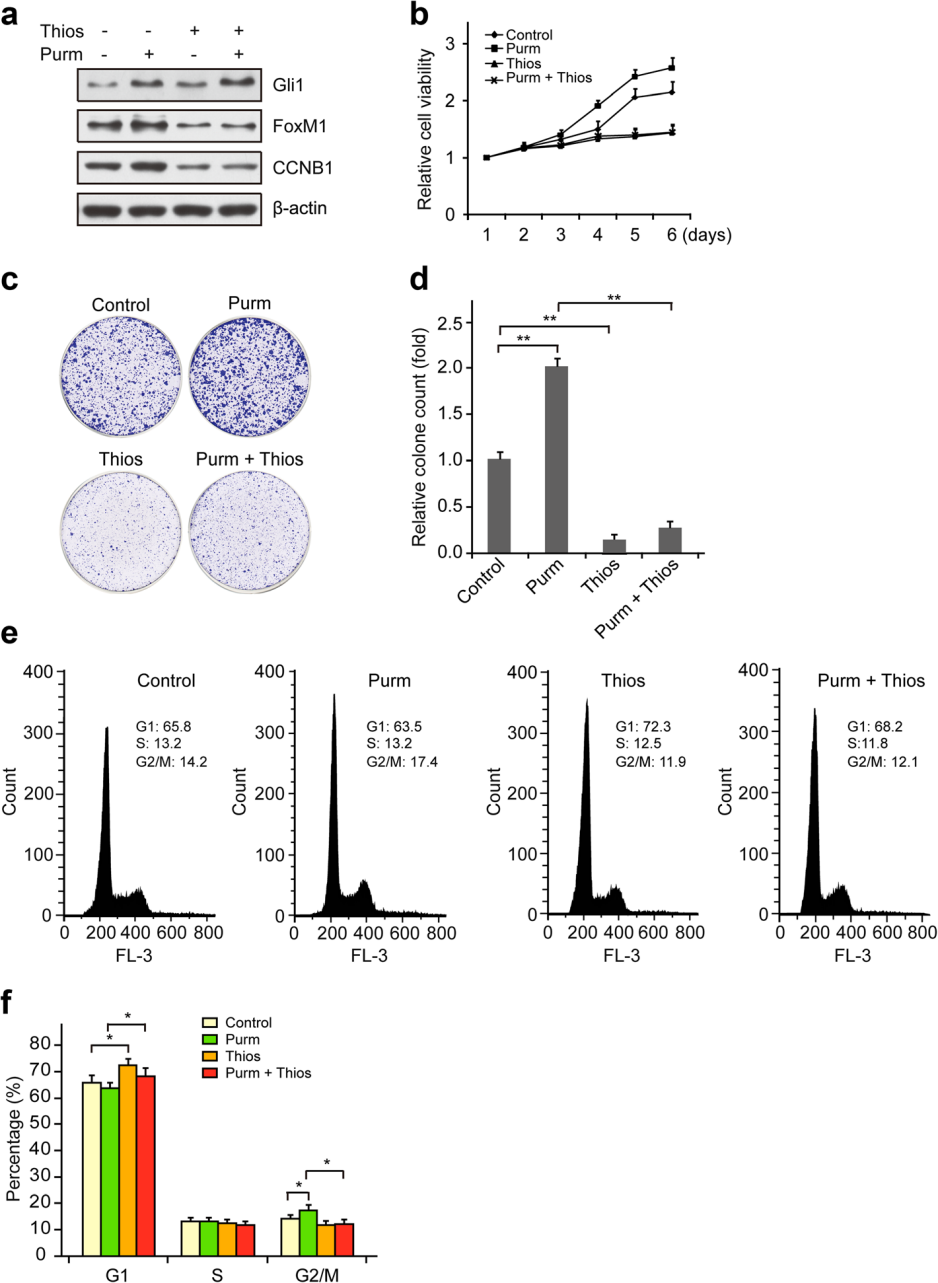
Gli1 regulates CRC cell proliferation depending on FoxM1. **a**-**d**) LoVo cells were separately or simultaneously treated with 1 μM purmorphamine and 1 μM thiostrepton for the indicated time. **a** The Gli1, FoxM1, and CCNB1 protein expression levels were examined by immunoblotting after drug treatment for 48 h. **b** Cell viability was detected after 6 days using an MTT assay. **c** LoVo cells treated with indicated drugs were cultured for 2 weeks, and outgrowth colonies were stained with crystal violet. **d** The matched colony count of (**c**). Error bars represent the mean and S.D. of three independent experiments. **, *p* < 0.01. **e** and **f** The cell cycle profile of LoVo cells was examined by fluorescence-activated cell sorting (FACS) with propidium iodine (PI) staining after 48 h of drug treatment (**e**), and the percentages of multinucleate cells were quantified and are shown as a histogram (**f**). Purm: purmorphamine; Thios: thiostrepton. *Error bars* represent the mean and S.D. of three independent experiments. *, *p* < 0.05

To further examine the necessity of FoxM1 expression for the Gli1-mediated proliferation of CRC cells, we performed complementary experiments by separately and simultaneously treating LoVo cells, which moderately expressed both endogenous FoxM1 and Gli1 proteins, with the Hh-Gli1 activator purmorphamine and the FoxM1 inhibitor thiostrepton [39]. As shown in Fig. 4a, purmorphamine upregulated the protein levels of Gli1, FoxM1 and CCNB1, whereas thiostrepton inhibited FoxM1 and CCNB1 expression in LoVo cells. Thiostrepton counteracted the activation effect of purmorphamine and led to the decreased expression of FoxM1 and CCNB1, but not of Gli1, when treating LoVo cells simultaneously. The results from the MTT assay showed that purmorphamine promoted LoVo cell viability compared to the control treatment, but thiostrepton inhibited cell viability, even when the cells were concurrently activated with purmorphamine (Fig. 4b). We next performed colony formation assays and a cell cycle analysis and found that thiostrepton also impeded the LoVo cell proliferation caused by purmorphamine, as evidenced by a significant reduction in colony number (Fig. 4c and d) and slower cell cycle progression, which was indicated by a higher fraction of cells in the G1 phase and a lower proportion of cells in the G2/M phase (Fig. 4e and f). Taken together, the results of these Gli1 activation and FoxM1 inhibition studies demonstrate that FoxM1 is required for Gli1-mediated CRC cell proliferation.

## Discussion

Previous studies have linked the Hh-Gli1 signaling pathway to numerous human cancers, including CRC [40]. However, the molecular mechanisms underlying the Hh-Gli1 signaling-mediated maintenance of CRC remain largely unclear. Here, we identified Hh-Gli1-FoxM1 as a new signaling axis in the proliferation of CRC and clarified this signaling axis pathway as a potential target for the future development of anti-CRC therapy.

Gli1, a transcriptional factor of the Hh signaling pathway, is upregulated in most digestive tumors, including pancreatic cancer, hepatocellular carcinoma and gastric cancer [41–44]. Similar results were also reported in other studies of CRC [12, 13, 45]. Although these studies found that the Gli1 mRNA and protein expression levels were significantly increased in CRC tissues, the exact mechanism underlying this increase remained unclear. The present study reports that FoxM1 activity is required for the Gli1-mediated promotion of CRC cell proliferation. Specifically, Gli1 ectopic overexpression using the Hh signaling pathway activator purmorphamine promoted CRC cell proliferation, whereas the simultaneous inhibition of FoxM1 with the FoxM1 inhibitor thiostrepton inhibited CRC cell proliferation. A very recent study indicates that Gli1 promotes CRC metastasis in a FoxM1-dependent manner by activating EMT and PI3K-AKT signaling [46], which is consistent with our results. However, we demonstrate that Gli1 promotes cell proliferation by directly binding to the promoter of FoxM1 and transactivating FoxM1 in CRC cells. The inhibition of Gli1 also slowed the progression from the G1 to the S phase, as evidenced by a cell cycle assay (Fig. 4e and f). In addition to promoting the proliferation of CRC cells, Gli1 mediated multiple aspects of cellular processes, including cell survival, invasion and metastasis [11, 45, 47, 48].

FoxM1 is a member of the forkhead box family of transcription factors and is involved in the control of cell proliferation, chromosomal stability, angiogenesis, and invasion. Increasing evidence has shown that FoxM1 expression is upregulated in many types of tumors [19, 21, 22, 49]. In this study, we found that FoxM1 expression was also elevated in CRC tumor tissues compared with the matched normal colorectal mucosa. Teh et al. suggested that FoxM1 is a downstream target of Gli1 in basal cell carcinomas [50], but their study lacked direct evidence. In the present study, we demonstrated that FoxM1 is a direct target of Gli1 using ChIP and luciferase reporter assays. Specifically, we identified one potential Gli1-binding site (GCCCACCCA) in the FoxM1 promoter, and the mutation of this site significantly attenuated the Gli1-mediated transactivation of FoxM1 promoters (Fig. 2b-d). Moreover, we found that the Hh-Gli1 signaling pathway regulated the expression of FoxM1 in CRC cells and that the inhibition of FoxM1 impeded Gli1-mediated CRC cell proliferation. FoxM1 expression was also recently reported to be modulated by many other transcription factors, and Her2 reportedly upregulated FoxM1 expression in gastric cancer [51]. FoxM1 was also shown to be transactivated by HSF1, which promoted the survival of glioma cells under heat shock stress [52]. An increasing number of studies have reported that FoxM1 mediates drug resistance in many types of cancers, including gastric cancer [53], breast cancer [54, 55] and glioblastoma [56], by regulating the expression of downstream targets. Together with these findings, our results suggest that the Hh-Gli1-FoxM1 axis can serve as a novel target for cancer therapy. Thus, further screening and validation of drugs that target Hh-Gli1-FoxM1 signaling would be interesting and significant.

## Conclusions

We reported herein that the Gli1 and FoxM1 expression levels are consistently elevated in human CRC tissues. Moreover, Gli1 regulates the transcription of FoxM1 by directly binding to the promoter of FoxM1 at BS4 (GCCCACCCA), which contributes to the proliferation of CRC cells. These observations uncover a novel molecular mechanism by which the Hh-Gli1-FoxM1 axis mediates CRC cell proliferation and provide a potential valid therapeutic target for the treatment of CRC in the future.

## Additional files

Additional file 1: Table S1. Primers used for real-time PCR amplification. (DOC 33 kb)
Additional file 2: Table S2. Primers used for ChIP. (DOC 30 kb)
Additional file 3: Figure S1. FoxM1 promotes the proliferation of CRC cells. (A and B) The validation of the FoxM1 overexpression plasmid in Caco2 cells (A) and interference plasmid in HCT116 cells (B), performance as indicated. (C and D) Overexpression of FoxM1 increased the colony formation and growth rate of Caco2 cells. (C) Caco2 cells transfected with the indicated plasmids were subjected to clonogenic assays. (D) Quantitative analysis of (C). (E and F) Knocking down FoxM1 reduced the colony formation and growth rate of HCT116 cells. (E) HCT116 cells transfected with the indicated plasmids were subjected to clonogenic assays. (F) Quantitative analysis of (E). (G and H) Overexpression of FoxM1 increased the proliferation rate of CaCo2 cells. (G) BrdU assay of the proliferation rate of CaCo2 cells transfected with the indicated plasmids for 48 h. (H) Quantitative analysis of (G). (I and J) Down-regulation of FoxM1 decreased the proliferation rate of HCT116 cells. (I) BrdU assay of the proliferation rate of HCT116 cells transfected with the indicated plasmids for 48 h. (J) Quantitative analysis of (I). All quantitative analyses were performed using the ImageJ software, and the error bars represent the mean and S.D. of three independent experiments. miR-FoxM1: miRNAi-FoxM1; miR-control: miRNAi-control. **, *p* < 0.01. (TIF 73489 kb)

## Abbreviations

BrdU: 5-bromo-2′-deoxyuridine
ChIP: Chromatin immunoprecipitation
CRC: Colorectal cancer
EDTA: Ethylenediamine tetraacetic acid
FACS: Fluorescence-activated cell sorting
FoxM1: Forkhead box M1
HCC: Hepatocarcinoma
Hh: Hedgehog
MTT: 3-(4, 5-dimethylthiazol-z-yl)-2, 5-diphenyltetrazolium bromide
PBS: Phosphate-buffered saline
PI: Propidium iodide
Ptch1: Patched-1
Smo: Smoothened
