# Supplementary material for: Aberrant activation of hedgehog signaling promotes cell proliferation via the transcriptional activation of forkhead Box M1 in colorectal cancer cells
Source: J Exp Clin Cancer Res. 2017 Feb 2;36:23. doi: 10.1186/s13046-017-0491-7 (PMC5288899; doi:10.1186/s13046-017-0491-7)
Supplement: Additional file 1: Table S1. — Primers used for real-time PCR amplification. (DOC 33 kb) [file 13046_2017_491_MOESM1_ESM.doc]

**Additional file 1:**

**Table S1. Primers used for real-time PCR amplification**

| **Genes** | **Forward primer (5’ to 3’)** | **Reverse primer (5’ to 3’)** |
| --- | --- | --- |
| Gli1 | 5’-TCCTACCAGAGTCCCAAGTT-3’ | 5’-CCCTATGTGAAGCCCTATTT-3’ |
| FOXM1 | 5’-TGCAGCTAGGATGTGAATCTTC-3’ | 5’-GGAGCCCAGTCCATCAGAACT-3’ |
| CCNB1 | 5’-CTCCCTGCTTCCTGTTATGC-3’ | 5’-TTCGACAACTTCCGTTAGCC-3’ |
| GAPDH | 5’-CAGGGCTGCTTTTAACTCTGGT-3’ | 5’-GATTTTGGAGGGATCTCGCT-3’ |
