# Supplementary material for: Aberrant activation of hedgehog signaling promotes cell proliferation via the transcriptional activation of forkhead Box M1 in colorectal cancer cells
Source: J Exp Clin Cancer Res. 2017 Feb 2;36:23. doi: 10.1186/s13046-017-0491-7 (PMC5288899; doi:10.1186/s13046-017-0491-7)
Supplement: Additional file 2: Table S2. — Primers used for ChIP. (DOC 30 kb) [file 13046_2017_491_MOESM2_ESM.doc]

**Additional file 2:**

**Table S2. Primers used for ChIP**

|  | **Predictive BS (5’ to 3’)** | **Forward primer (5’ to 3’)** | **Reverse primer (5’ to 3’)** |
| --- | --- | --- | --- |
| FoxM1-BS1 | 5’-GGGACCCCCTACA-3’ | 5’-TGCCAACACTGTGAAAGG-3’ | 5’-CACTACAACCTCAACCTCC-3’ |
| FoxM1-BS2 | 5’-CTGACAACCCCCC-3’ | 5’-TTGTAGTGAGCCAAGGTC-3’ | 5’-ACACTATTCCCTGACCAA-3’ |
| FoxM1-BS3 | 5’-TCTACCTCCCATC-3’ | 5’-ATCATTCCCCTGACAACC-3’ | 5’-GAGAAAGAAGGAGACAGAG-3’ |
| FoxM1-BS4 | 5’-TCGCCCACCCACG-3’ | 5’-CGAGCTTTGAAAAGGGGAGC-3’ | 5’-GGAAGAAGTGGCCGTGGG-3’ |

BS: Binding Sequences.
